# Supplementary material for: Effect of Mandibular Advancement Device Treatment on the Site-Specific Degree of Upper Airway Collapse During Drug-Induced Sleep Endoscopy
Source: J Clin Med. 2025 Nov 17;14(22):8142. doi: 10.3390/jcm14228142 (PMC12653495; doi:10.3390/jcm14228142)
Supplement: Supplementary file 1 [file jcm-14-08142-s001.zip › jcm-3906934-supplementary.pdf]

Supplementary material

Effect of mandibular advancement device treatment on the site-specific degree of upper airway collapse during drug-induced sleep endoscopy

Eldar Tukanov[1,2,*], Marijke Dieltjens[1,2], Annelies E. Verbruggen[2], Anneclaire V. Vroegop[1,2,3], Johan A. Verbraecken[3,4,5], Paul H. Van de Heyning[1,2], Marc J. Braem[1,2], Sara Op de Beeck[1,2], Olivier M. Vanderveken[1,2,3]

[1] Translational Neurosciences, Faculty of Medicine and Health Sciences, University of Antwerp, 2610 Wilrijk, Belgium
[2] Department of ENT, Head and Neck Surgery, Antwerp University Hospital, 2650 Edegem, Belgium
[3] Multidisciplinary Sleep Disorders Centre, Antwerp University Hospital, 2650 Edegem, Belgium
[4] Department of Pulmonary Medicine, Antwerp University Hospital, 2650 Edegem, Belgium
[5] Research Group LEMP, Faculty of Medicine and Health Sciences, University of Antwerp, 2610 Wilrijk, Belgium
* Correspondence: eldar.tukanov@uantwerpen.be

Table S1 – Clinical and demographical characteristics of population at baseline. Subdivision in mild, moderate and severe OSA.

	Total (n = 69)	Mild OSA: AHI < 15 (n = 34) †, ‡	Moderate OSA: 15 ≥ AHI <30 (n = 28)	Severe OSA: 30 ≥ AHI < 50 (n = 7)	p-value
Sex (male/female; n)	59/10	28/6	24/4	7/0	.698[a]
Age (years)	48.4 ± 9.5	48.7 ± 11.1	48.3 ± 8.4	47.1 ± 5.8	.923[b]
BMI (kg/m^2)	27.5 ± 3.1	26.7 ± 3.3	27.8 ± 2.6	30.2 ± 1.9	.014[b]
VAS (0 – 10) snoring	6.0 (5.0 – 9.0)	6.0 (5.0 – 9.0) ‡	6.0 (5.3 – 9.0)	9.0 (6.0 – 10.0)	.290[c]
ESS (0 – 24)	7.0 (5.0 – 13.5)	7.5 (4.0 – 11.0)	7.0 (5.0 – 14.5)	14.0 (6.0 – 16.0)	.255[c]
AHI (events/h)	16.5 (11.1 – 23.5)	11.1 (7.3 – 12.8)	22.0 (18.7 – 25.0)	34.6 (34.0 – 40.7)	<.001[c]
Supine AHI (events/h)	29.4 (18.3 – 52.5)	20.7 (12.3 – 36.0)	39.0 (27.0 – 53.4)	45.9 (39.3 – 83.3)	.006[c]
Non-supine AHI (events/h)	8.9 (4.2 – 16.5)	5.9 (3.0 – 8.7)	14.3 (9.3 – 19.7)	20.9 (17.7 – 36.9)	<.001[c]
ODI (events/h)	4.4 (2.4 – 10.9)	2.6 (1.2 – 4.5)	8.3 (3.4 – 11.4)	16.6 (15.5 – 23.7)	<.001[c]
Mean SaO_2 (%)	95.1 (94.1 – 96.1)	95.5 (94.4 – 96.4)	94.6 (93.7 – 95.7)	94.9 (93.3 – 96.1)	.083[c]
Minimal SaO_2 (%)	87.0 (84.0 – 90.0)	89.0 (84.6 – 91.0) †	86.0 (84.7 – 89.2)	83.0 (79.0 – 86.0)	.028[c]

Abbreviations: AHI (apnea–hypopnea index); BMI (body mass index); ESS (Epworth sleepiness scale); ODI (oxygen desaturation index); OSA (obstructive sleep apnea); SaO_2 (oxygen saturation); VAS (visual analogue scale for snoring).

Note: Data are presented as median (quartile 1–quartile 3) for non-normally distributed data or mean ± SD for normally distributed data. AHI was scored according to the American Academy of Sleep Medicine 1999 criteria (3% oxygen desaturation or an arousal). ODI was calculated as dips of ≥3% over the total time in bed. † Two participants with AHI < 15 had no minimal saturation data available. ‡ One participant with AHI < 15 had no VAS score available. [a] Fisher-Freeman-Halton Exact test; [b] ANOVA (analysis of variance); [c] Kruskal-Wallis test; P values compare the three subgroups.

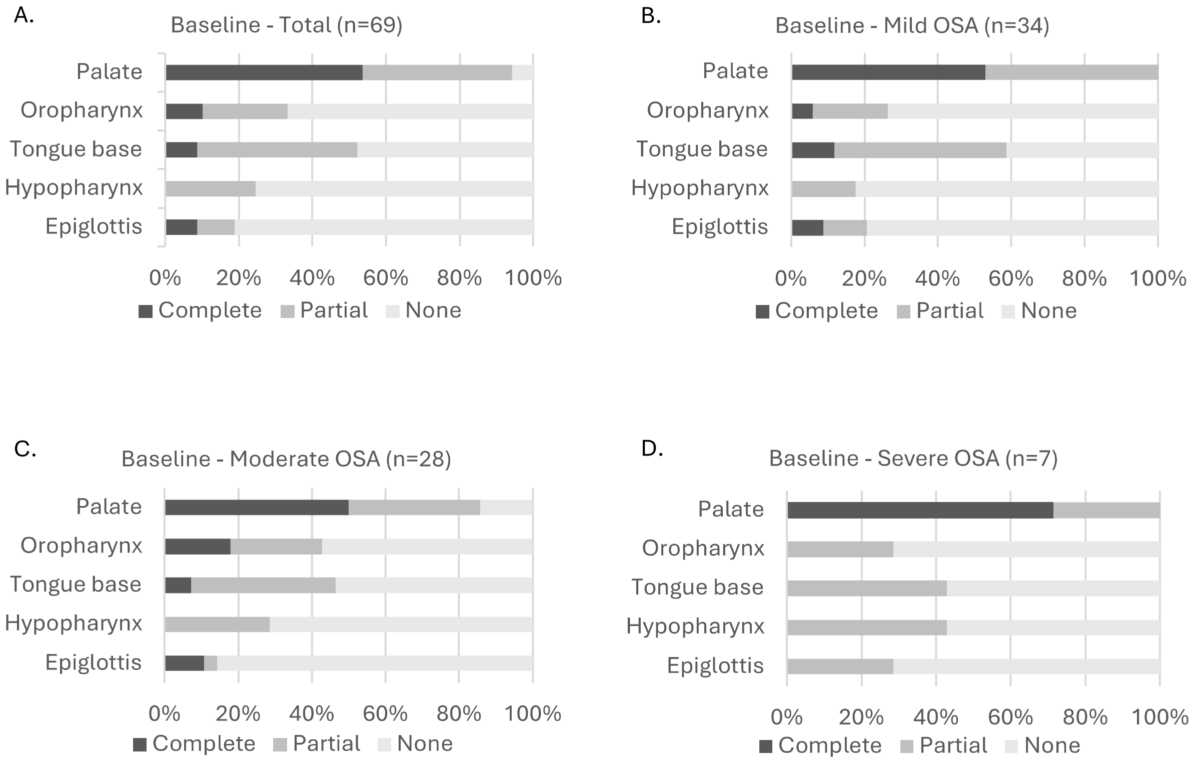

Figure S1 – Distribution of collapse degree at each upper airway level at baseline. A: Collapse in all participants; B: Collapse in participants with mild OSA; C: Collapse in participants with moderate OSA; D: Collapse in participants with severe OSA. Fisher-Freeman-Halton Exact test showed no significant differences in collapse degree distribution across OSA severity subgroups (panel B-D). Abbreviations: OSA (obstructive sleep apnea)

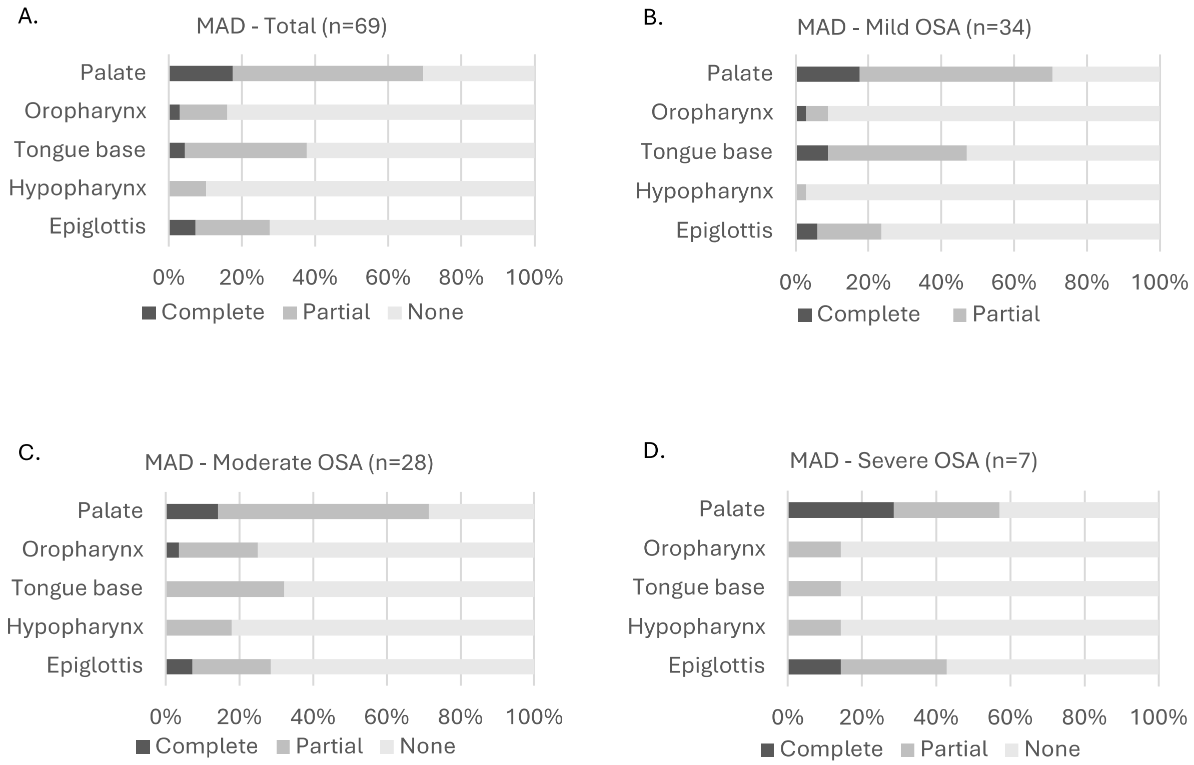

Figure S2 – Distribution of collapse degree at each upper airway level with MAD. A: Collapse in all participants; B: Collapse in participants with mild OSA; C: Collapse in participants with moderate OSA; D: Collapse in participants with severe OSA. Abbreviations: MAD (mandibular advancement device); OSA (obstructive sleep apnea).

Table S2 – Exploratory logistic regression analysis on the change of site-specific upper airway collapse degree from baseline to MAD therapy, for each OSA severity group. Without and with correction for BMI and AHI.

**Mild OSA: AHI < 15 (n = 34)**				
Site of collapse	Ordinal logistic regression		+ BMI + AHI	
	p	OR (95% CI)	p	OR (95% CI)
Palate	<.001	7.70 (3.18; 18.64)	<.001	7.78 (3.18; 19.06)
Oropharynx	.035	3.63 (1.09; 12.05)	.035	3.64 (1.09; 12.11)
Tongue base	.034	1.56 (1.03; 2.37)	.035	1.69 (1.04; 2.75)
Hypopharynx	.038	7.07 (1.11; 44.89)	.044	7.25 (1.06; 49.65)
Epiglottis	.664	.89 (.52; 1.52)	.755	.92 (.52; 1.60)
**Moderate OSA: 15 ≥ AHI <30 (n = 28)**				
Site of collapse	Ordinal logistic regression		+ BMI + AHI	
	p	OR (95% CI)	p	OR (95% CI)
Palate	.001	4.12 (1.74; 9.77)	.002	4.16 (1.72; 10.02)
Oropharynx	.024	2.49 (1.13; 5.50)	.010	2.81 (1.28; 6.16)
Tongue base	.010	1.99 (1.18; 3.36)	.010	1.98 (1.18; 3.33)
Hypopharynx	.071	1.84 (.95; 3.57)	.010	3.06 (1.31; 7.13)
Epiglottis	.082	.48 (.21; 1.10)	.082	.47 (.20; 1.10)
**Severe OSA: 30 ≥ AHI < 50 (n = 7)**				
Site of collapse	Ordinal logistic regression		+ BMI + AHI	
	p	OR (95% CI)	p	OR (95% CI)
Palate	.004	9.06 (2.02; 40.65)	.016	33.05 (1.91; 571.33)
Oropharynx	.295	2.40 (.47; 12.37)	.296	2.40 (.47; 12.40)
Tongue base	.127	4.50 (.65; 31.08)	.092	4.60 (.78; 27.18)
Hypopharynx	.127	4.50 (.65; 31.08)	.113	4.87 (.69; 34.44)
Epiglottis	.248	.46 (.12; 1.73)	.140	.31 (.07; 1.47)

Abbreviations: AHI (apnea-hypopnea index); BMI (body mass index); OR (odds ratio). Note: P-values and OR were calculated with a confidence interval of 95%. Significant values ($p < 0.05$) are shown in bold.

Table S3 – Exploratory logistic regression analysis on the change of site-specific upper airway collapse degree from baseline to MAD therapy using the VOTE classification (oropharynx and hypopharynx combined) in the total population. Without and with correction for BMI and AHI.

**Total (n = 69)**				
Site of collapse	Ordinal logistic regression		+ BMI + AHI	
	p	OR (95% CI)	p	OR (95% CI)
Palate	<.001	7.70 (3.18; 18.64)	<.001	7.78 (3.18; 19.06)
Oro-/hypopharynx	<.001	3.66 (2.05; 6.56)	<.001	3.91 (2.15; 7.12)
Tongue base	.034	1.56 (1.03; 2.37)	.035	1.69 (1.04; 2.75)
Epiglottis	.664	.89 (.52; 1.52)	.755	.92 (.52; 1.60)

Abbreviations: AHI (apnea-hypopnea index); BMI (body mass index); OR (odds ratio). Note: P-values and OR were calculated with a confidence interval of 95%. Significant values ($p < 0.05$) are shown in bold.

Table S4 – Exploratory logistic regression analysis on the change of site-specific upper airway collapse degree from baseline to MAD therapy using the VOTE classification (oropharynx and hypopharynx combined), for each OSA severity group (moderate and severe OSA group combined). Without and with correction for BMI and AHI.

**Mild OSA: AHI < 15 (n = 34)**				
Site of collapse	Ordinal logistic regression		+ BMI + AHI	
	p	OR (95% CI)	p	OR (95% CI)
Palate	<.001	7.70 (3.18; 18.64)	<.001	7.78 (3.18; 19.06)
Oro-/hypopharynx	.013	4.771 (1.39; 16.34)	.011	4.88 (1.43; 16.66)
Tongue base	.034	1.56 (1.03; 2.37)	.035	1.69 (1.04; 2.75)
Epiglottis	.664	.89 (.52; 1.52)	.755	.92 (.52; 1.60)
**Moderate-to-severe OSA: 15 ≥ AHI <50 (n = 35)**				
Site of collapse	Ordinal logistic regression		+ BMI + AHI	
	p	OR (95% CI)	p	OR (95% CI)
Palate	.001	4.12 (1.74; 9.77)	.002	4.16 (1.72; 10.02)
Oro-/hypopharynx	<.001	3.59 (1.82; 7.08)	<.001	4.09 (2.12; 7.91)
Tongue base	.010	1.99 (1.18; 3.36)	.010	1.98 (1.18; 3.33)
Epiglottis	.082	.48 (.21; 1.10)	.082	.47 (.20; 1.10)

Abbreviations: AHI (apnea-hypopnea index); BMI (body mass index); OR (odds ratio). Note: P-values and OR were calculated with a confidence interval of 95%. Significant values ($p < 0.05$) are shown in bold.

Table S5 – Exploratory logistic regression analysis on the change of site-specific upper airway collapse degree from baseline to MAD therapy using the VOTE classification (oropharynx and hypopharynx combined), for each OSA severity group (moderate and severe OSA group seperate). Without and with correction for BMI and AHI.

**Mild OSA: AHI < 15 (n = 34)**				
Site of collapse	Ordinal logistic regression		+ BMI + AHI	
	p	OR (95% CI)	p	OR (95% CI)
Palate	<.001	7.70 (3.18; 18.64)	<.001	7.78 (3.18; 19.06)
Oro-/hypopharynx	.013	4.771 (1.39; 16.34)	.011	4.88 (1.43; 16.66)
Tongue base	.034	1.56 (1.03; 2.37)	.035	1.69 (1.04; 2.75)
Epiglottis	.664	.89 (.52; 1.52)	.755	.92 (.52; 1.60)
**Moderate OSA: 15 ≥ AHI <30 (n = 28)**				
Site of collapse	Ordinal logistic regression		+ BMI + AHI	
	p	OR (95% CI)	p	OR (95% CI)
Palate	.001	4.12 (1.74; 9.77)	.002	4.16 (1.72; 10.02)
Oro-/hypopharynx	.002	3.13 (1.54; 6.38)	<.001	3.72 (1.86; 7.45)
Tongue base	.010	1.99 (1.18; 3.36)	.010	1.98 (1.18; 3.33)
Epiglottis	.082	.48 (.21; 1.10)	.082	.47 (.20; 1.10)
**Severe OSA: 30 ≥ AHI < 50 (n = 7)**				
Site of collapse	Ordinal logistic regression		+ BMI + AHI	
	p	OR (95% CI)	p	OR (95% CI)
Palate	.004	9.06 (2.02; 40.65)	.016	33.05 (1.91; 571.33)

Oro-/hypopharynx	.054	8.00 (.96; 66.45)	.054	8.02 (.96; 66.89)
Tongue base	.127	4.50 (.65; 31.08)	.092	4.60 (.78; 27.18)
Epiglottis	.248	.46 (.12; 1.73)	.140	.31 (.07; 1.47)

Abbreviations: AHI (apnea-hypopnea index); BMI (body mass index); OR (odds ratio). Note: P-values and OR were calculated with a confidence interval of 95%. Significant values ($p < 0.05$) are shown in bold.
